# Electrode-assisted acetoin production in a metabolically engineered *Escherichia coli* strain

**DOI:** 10.1186/s13068-017-0745-9

**Published:** 2017-03-14

**Authors:** Andreas H. Förster, Sebastian Beblawy, Frederik Golitsch, Johannes Gescher

**Affiliations:** 1 Department of Applied Biology, Institute for Applied Biosciences, Karlsruhe Institute of Technology, Fritz-Haber-Weg 2, 76131 Karlsruhe, Germany; 2 Department of Microbiology of Natural and Technical Interfaces, Institute of Functional Interfaces, Karlsruhe Institute of Technology, Hermann-von-Helmholtz-Platz 1, 76344 Eggenstein-Leopoldshafen, Germany

**Keywords:** Electrode-assisted fermentation, *Escherichia coli*, Bulk chemicals, Acetoin, Metabolic engineering

## Abstract

**Background:**

This paper describes the metabolic engineering of *Escherichia coli* for the *anaerobic* fermentation of glucose to acetoin. Acetoin has well-established applications in industrial food production and was suggested to be a platform chemical for a bio-based economy. However, the biotechnological production is often hampered by the simultaneous formation of several end products in the absence of an electron acceptor. Moreover, typical production strains are often potentially pathogenic. The goal of this study was to overcome these limitations by establishing an electrode-assisted fermentation process in *E. coli*. Here, the surplus of electrons released in the production process is transferred to an electrode as anoxic and non-depletable electron acceptor.

**Results:**

In a first step, the central metabolism was steered towards the production of pyruvate from glucose by deletion of genes encoding for enzymes of central reactions of the anaerobic carbon metabolism (Δ*frdA*-*D* Δ*adhE* Δ*ldhA* Δ*pta*–*ack*). Thereafter, the genes for the acetolactate synthase (*alsS*) and the acetolactate decarboxylase (*alsD*) were expressed in this strain from a plasmid. Addition of nitrate as electron acceptor led to an anaerobic acetoin production with a yield of up to 0.9 mol acetoin per mol of glucose consumed (90% of the theoretical maximum). In a second step, the electron acceptor nitrate was replaced by a carbon electrode. This interaction necessitated the further expression of *c*-type cytochromes from *Shewanella oneidensis* and the addition of the soluble redox shuttle methylene blue. The interaction with the non-depletable electron acceptor led to an acetoin formation with a yield of 79% of the theoretical maximum (0.79 mol acetoin per mol glucose).

**Conclusion:**

Electrode-assisted fermentations are a new strategy to produce substances of biotechnological value that are more oxidized than the substrates. Here, we show for the first time a process in which the commonly used chassis strain *E. coli* was tailored for an electrode-assisted fermentation approach branching off from the central metabolite pyruvate. At this early stage, we see promising results regarding carbon and electron recovery and will use further strain development to increase the anaerobic metabolic turnover rate.

**Electronic supplementary material:**

The online version of this article (doi:10.1186/s13068-017-0745-9) contains supplementary material, which is available to authorized users.

## Background

**Fig. 1:**
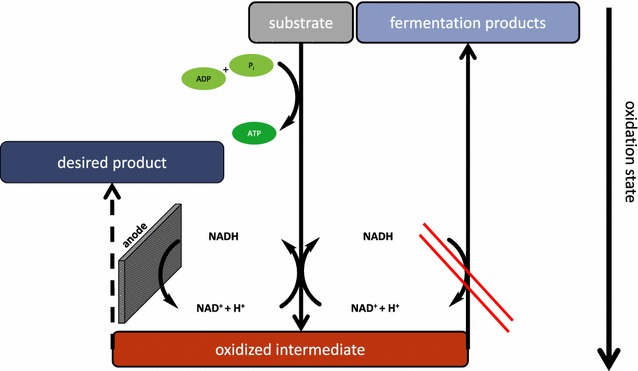
Scheme of an unbalanced fermentation. An unbalanced fermentation integrates two elements: First, the metabolism of a production organism is engineered in such a way, that only one desired substance is produced. As a consequence, redox equivalents like NAD^+^ cannot be regenerated anymore. The second element is the bioelectrochemical reactor supplying the organisms with an anode as a non-depletable electron acceptor

The envisioned socioeconomical migration towards a bioeconomy necessitates the development of new approaches for the production of bulk and fine chemicals. Electrode-assisted fermentations are a promising new tool for biotechnological production processes. They offer the possibility to gain single fermentation products that are more oxidized than the substrate under anoxic conditions [1, 2]. The catalyst of electrode-assisted fermentations is a composite material of a microorganism as biocatalyst and an electrode that acts as solid state respiratory electron acceptor. The engineering of the metabolism towards the production of an end product being more oxidized than the substrate leads to the release of a surplus of electrons which can be transferred to the electrode. The technology offers the possibility to combine the efficiency of anoxic processes with the production of a wide spectrum of single end products because the formation of side-products to balance the oxidation state is not necessary anymore (Fig. 1). The transfer of electrons from a microorganism to an electrode necessitates an extended respiratory chain to the cell surface. *Shewanella oneidensis* and *Geobacter sulfurreducens* are the best studied model organisms regarding electrode-assisted respiration [3]. Recently, *Escherichia coli* strains that are capable of electrode respiration have been developed and it was shown that the integration of synthetic electron transfer chains leading to an electrode can effectively change the anaerobic product spectrum of *E. coli* strains towards more oxidized end products [2, 4, 5]. This ability is based on the expression of genes for elements of the electron transport chain to the cell surface from *S. oneidensis*. The transfer of electrons to the electrode surface seems to be limited in *S. oneidensis* by the concentration of a three protein complex (MtrABC) in the outer membrane [6]. Expression of this protein complex in *E. coli* together with other compounds of the *S. oneidensis* electron transfer chain did not lead to productivities comparable to *S. oneidensis* so far [7, 8]. Hence, Sturm-Richter et al. successfully used the membrane permeable electron shuttle methylene blue to bridge the electron transfer gap through the outer membrane [2].

Acetoin is a bulk chemical of industrial interest. It is one example for substances that cannot be produced as sole end product in an anaerobic fermentation because it is more oxidized than typical substrates as for instance glucose. It occurs under environmental conditions as an intermediate of butanediol fermentation in *Bacillus subtilis* and several *Enterobacteria*. Acetoin has a well-established application as food additive, due to its intense butter flavor and is also a precursor for the synthesis of pyrazine flavor compounds. The average daily consumption per person is between 2 and 4 mg. Hence, the global annual volume is in the range of several thousand tons [9]. Interestingly, Werpy et al. ranked acetoin within the 30 most promising bio-based building block precursors for a petro-independent economy [10]. It is produced from pyruvate in a two-step reaction. At first, the acetolactate synthase (AlsS) catalyzes the condensation of two molecules of pyruvate to acetolactate. One molecule of CO_2_ is released in this reaction. In the second step, acetolactate is decarboxylated by the acetolactate decarboxylase (AlsD) [11–13]. Only low acetoin yields can be achieved using wild-type strains, since several other products are formed—foremost 2, 3-butanediol. The relative amounts depend highly on the NADH level in the cytoplasm and consequently at least partly on the relative abundance of an electron acceptor [14, 15]. To gain larger quantities of acetoin, several attempts were made with genetically engineered organisms. *Escherichia coli* is a common choice as a host organism, due to its versatile metabolism and genetic tractability [16–18]. *Bacillus subtilis*, *Serratia marcescens*, *Clostridium acetobutylicum,* and *Candida glabrata* have also been investigated as production strains [19–22]. The reported yields in different studies for an aerobic acetoin production in *E. coli* were around 80% of the theoretical maximum (Table 1). In contrast, studies under anoxic growth conditions resulted only in low yields of acetoin [16, 23]. However, oxic processes are typically less favorable for biotechnological bulk chemical production, due to the necessity of aeration and the higher ratio of anabolism over catabolism [24]. At least the latter limitation can be tackled by a very promising approach, which was also used in several further *E. coli* strain developments aiming at the production of pyruvate [25–27]. The authors deleted the proton translocating part of the membrane-bound ATPase and the *poxB* gene, encoding pyruvate oxidase [28]. This led to a strain that could use oxygen as electron acceptor but was not able to conduct energy production based on oxidative phosphorylation.

**Table 1:** Acetoin production in several approaches

Substrate	Yield (%)	Conditions	Complex compounds	Reference
*E. coli*				
Glucose	90	Anoxic, NO_3_^−^		This study
Diacetyl/glucose	88	Oxic	LB	[53]
Glucose	80	Oxic	Yeast extract	[17]
Glucose	96	Oxic	LB	[51]
Glucose	49	Oxic	LB	[54]
*S. oneidensis*				
Lactate	86	Anoxic, fumarate		[29]
*B. subtilis*				
Glucose/xylose	75	Oxic		[52]
Wood hydrolysate	69.5	Oxic		[22]
Glucose	83.5	Oxic	Beef extract	[55]

Recently, Bursac et al. studied the electrode-assisted fermentation based production of acetoin by using *S. oneidensis* as biocatalyst [29]. The authors used lactate as carbon and electron source and achieved a production of 78% of the theoretical production maximum. Nevertheless, the spectrum of usable carbon sources is rather narrow for *S. oneidensis*. Sufficient growth under anoxic conditions is only sustained with lactate, while typical cheap substrates as for instance glucose or glycerol cannot be anaerobically metabolized.

In this study, an *E. coli* strain was developed as biocatalyst for an electrode-assisted acetoin production. Hence, the impact of the deletion of four genes encoding enzymes of the mixed acid fermentation pathways was investigated. A high efficient chassis strain from the characterization routine was developed further to establish acetoin production in an electrode-assisted fermentation.

## Methods

### Plasmid and strain construction

Plasmids were constructed using standard methods [30]. Primers and plasmids used in this study are listed in Additional file 1: Table S1 and Additional file 2: Table S2. Strains applied in this study are found in Additional file 3: Table S3. All deletions were constructed in strain JG146 which contains a synthetic operon encoding for *cymA* and *mtrA* in the genome. Integration of the *cymA*–*mtrA* fragment caused a deletion of the *E. coli frd* gene locus [31]. The deletion of *adhE* was performed as described before [32]. The deletion fragment was obtained with primers 1 and 2 using pSG76–CSH as a template. The amplificate was transformed into *E. coli*, carrying the plasmid pKD46 and integrated in the genome as described in [33]. Cells were spread on LB-agar plates containing chloramphenicol (6–10 µg/ml). Clones were tested by PCR with primers 3 and 4. The chloramphenicol resistance cassette was deleted as described by [32]. For the construction of the *ldhA*-deletion plasmid, genes were amplified by PCR and cloned in the host vector in four steps. The first step was the ligation of *cscRAKB_LguI* in the linearized (*Sma*I) vector pASK43+ leading to plasmid pASK43+ *_csc_LguI*. The *cscRAKB_LguI* fragment was obtained from a PCR using primers 5 and 6 and plasmid pKJL124 as template [34]. Secondly, plasmid pASK43+ *_csc_LguI* was linearized (*Lgu*I) and ligated with the PCR product of primers 7 and 8 which comprises the upstream homologous region to *ldhA* (pASK43+ *_csc_ldh*-*up*). Thirdly, the resulting plasmid was used as the template for the reaction with primers 9 and 10. The PCR product was then ligated in the *Sma*I-digested pASK43+ vector (pASK43+ *LguI_csc_ldh*-*up*). In the last construction step, the plasmid was linearized using the *Lgu*I sites added with primer 9 and ligated with the PCR product of primer 11 and 12 comprising the downstream homologous region. The plasmid was digested with *Xba*I resulting in the final deletion fragment containing homologous regions to the up- and downstream areas of *ldhA* flanking the *csc*-genes for the sucrose utilization- based selection (pASK43+ *_ldh*-*down_csc_ldh*-*up*). It was used to delete *ldhA* in the Δ*frd* Δ*adhE* mutant via homologous recombination mediated by pKD46. Deletion of *pta*–*ack* was performed as described by [35]. First, *galK* was amplified using the primers 13 and 14 (template JG287) and the first homologous region was amplified via PCR with primer 15 and 16 using genomic DNA of *E. coli* as template. Primer 13 and 16 were phosphorylated. The fragment containing *galK* was digested with *Hind*III and the fragment including the homologous upstream region was cut with *Nhe*I. After the ligation into the H*ind*III and *Nhe*I-digested pASK43+ vector, the product was linearized with *Hind*III and dephosphorylated (alkaline phosphatase, Thermo Fisher Scientific). The second fragment, necessary for homologous recombination, was amplified with the primers 17 and 18. It was integrated in the vector using isothermal in vitro ligation as described by Gibson et al. [36]. The constructed plasmid and the helper plasmid pACBSR [35] were used for the transformation in *E. coli*. After induction of pACBSR with arabinose (final concentration 0.2%), cells were plated on M63-agar with galactose as sole carbon source and tested via colony-PCR (Primer 19 and 20).

The production of acetoin requires the activity of acetolactate synthase (*alsS*) and acetolactate decarboxylase (*alsD*). Therefore, the plasmid pMAL_*alsSD* was designed for acetoin production. The insert was obtained using *Bacillus subtilis* PY79 as template and the primers 21 and 22 for amplification. The amplificate was then ligated into a pMAL vector.

*Stc* was integrated into the genome using the CRIM-System [37]. The plasmid was constructed and integrated at the phage attachment site P21 as described by [2]. The pEC86 plasmid containing the *ccm* genes of *E. coli* was transformed into the production strain, to improve the expression of *c*-type-cytochromes [38].

### Growth conditions and media

*Escherichia coli* was routinely grown in LB medium [1% (w/v) yeast extract, 0.5% (w/v) NaCl, 0.5% (w/v) peptone]. Media were supplemented with antibiotics, if necessary. For the selection of the *galK* insertion, *E. coli* was grown on M63 agar plates [25 mM (NH_4_)_2_SO_4_, 100 mM KH_2_PO_4_, 9 µM FeSO_4_, 0.1 mM CaCl_2_, 1 mM MgSO_4_, thiamine [0.05‰ (w/v)], and agar [2% (w/v)], pH 7] with galactose (0.2%) as sole carbon source. All growth experiments were conducted in independent triplicates. Oxic growth experiments were performed in flasks containing M9 medium (47.6 mM Na_2_HPO_4_, 22 mM KH_2_PO_4_, 18.6 mM NH_4_Cl, 8.6 mM NaCl_2_, 10 mM HEPES) complemented with trace elements (100×: 0.27 mM CoCl_2_, 0.02 mM CuSO_4_, 5.66 mM H_3_BO_3_, 0.24 mM Fe(II)Cl_2_, 6.72 mM Na_2_-EDTA, 0.13 mM MnSO_4_, 0.22 mM Na_2_MoO_4_, 0.13 mM Na_2_SeO_4_, 1 mM NaCl, 0.5 mM NiCl, 0.16 ZnSO4), CaCl_2_ (final concentration: 0.1 mM), MgSO_4_ (1 mM), casamino acids [0.15% (w/v)], thiamine [0.05‰ (w/v)], and glucose (10 mM). Anoxic growth experiments were conducted in N_2_-flushed culture flasks. Under anoxic respiratory conditions, DMSO (final concentration: 50 mM) or nitrate (final concentration: 12.5 mM) was added to the medium. Culture flasks were inoculated to an initial optical density (600 nm) of 0.05. Cultures were incubated at 30 °C in a rotary shaker (125 rpm) for 24–96 h. Samples were taken at the beginning and the end of the experiment for end product analysis.

Acetoin production experiments were conducted anoxically in sealed flasks containing M9–medium. Expression of the *als*-genes was induced with 50 µM IPTG.

For the bioelectrochemical approach, cells were cultured in 20 ml aerobic LB medium supplemented with antibiotics (10 µg/l kanamycin sulfate, 100 µg/l ampicillin, 50 µg/l chloramphenicol) overnight at 37 °C. Afterwards, the cells were transferred to 500 ml sterile LB medium with identical supplementation and again incubated over night at 37 °C. The gained cell mass was harvested by centrifugation at 6000×*g* for 8 min and resuspended in an equal volume of fresh LB medium. In addition, this culture was supplemented with 14.8 µM anhydrotetracycline for the induction of the *cymA*–*mtrA* locus and 1 mM arabinose for the induction of *stc*. The induction period took at least 4 h. Thereafter, the cells were washed twice and then resuspended in washing buffer [6]. The cells were then transferred to 200 ml of an anoxic phosphate-buffered minimal medium supplemented with 50 µM methylene blue and the necessary inducers and antibiotics [6]. The carbon source was 20 mM glucose. The initial OD_600_ was adjusted to 6.

### Electrode-assisted fermentation experiments

**Fig. 2:**
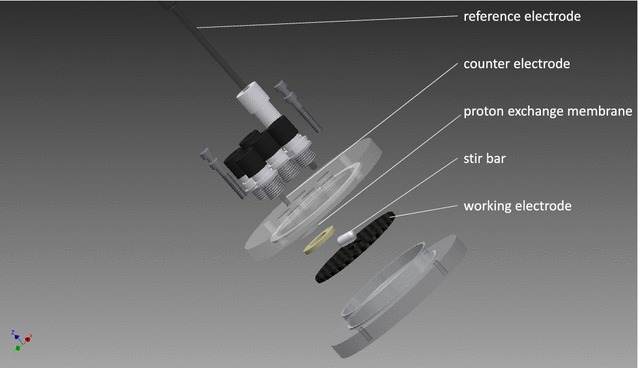
Schematic image of the bioelectrochemical reactor. The reactor setup had a volume of 23 ml and a 40 cm^2^ working electrode placed at the *bottom*. The polycarbonate lid provided ports for sampling the reference electrode as well as the counter electrode compartment. This design comprised a high surface-to-volume ratio with minimal distance between the electrodes

A bioelectrochemical system was developed that had a volume of 23 ml. The reactor consisted of a three-electrode-setup, with a ring-shaped graphite felt (40 cm^2^ surface, GFD 2.5, Sigracell, Germany) as the working electrode. This electrode was placed at the bottom and a stir bar was placed in the center of the reactor, which was not covered by the electrode (Fig. 2). An Ag/AgCl electrode (Xylem group, Germany) was used as reference and a platinum mesh (1.25 cm^2^, chemPUR, Germany) as the counter electrode. Working and counter electrode were separated through a proton exchange membrane (Fumapem F-950, FUMATECH, Germany). The electrode material was soaked in sterile reactor medium and was preincubated at an applied potential of +200 mV vs. normal hydrogen electrode overnight. The preincubation medium was removed from the reactor before the reactor volume was filled with inoculated medium as stated above. To provide strict anoxic conditions, the reactor was placed in an anaerobic chamber (Coy Laboratory Products, USA) with an N_2_/H_2_ atmosphere (98%/2%). During the chronoamperometric experiment, the working electrode was poised to +200 mV vs. normal hydrogen electrode, and the electrical current was measured using a potentiostat (PalmSens, Netherlands or Pine instruments, USA). As a control, analogous open-circuit potentiometry experiments were conducted. Coulombic efficiencies of the chronoamperometric experiments have been calculated according to Kipf et al. based on substrate conversion and product formation [39].

### Analytical methods

Glucose and formic acid were determined enzymatically. Glucose was quantified using hexokinase and glucose-6-phosphate dehydrogenase. A sample volume of 20 µl was mixed with 180 µl of the reaction buffer (0.75 M triethanolamine, 10 mM Mg^2+^, 1.1 mM NADP^+^, 8 mM ATP) containing the enzymes hexokinase (2.1 U/ml) and glucose-6-phosphate dehydrogenase (2.1 U/ml). The absorbance at 340 nm was determined after incubation at 37 °C for 20 min, and the glucose concentration was calculated based on a calibration curve. Formic acid determination was conducted in a 96-well assay with standard concentrations as a reference. Each well was filled with 20 µl sample and 190 µl reaction buffer (final concentration in the assay: 50 mM potassium phosphate buffer pH 7.5, 1 mM pyrazole, 6.3 mM NAD^+^, and 3.6 U/ml formate-dehydrogenase). Ethanol was quantified via gas chromatography (Clarus 480, Perkin Elmer). The measurement was performed using a ZB-WAXplus column (Phenomenex) and nitrogen as the carrier gas (2.3 ml/min flow at the detector). Gas chromatography parameters (synthetic air flow: 5.5 bar, oven temperatures: 40 °C for 5 min, 5 °C/min up to 150 °C, hold 150 °C for 5 min, 20 °C/min up to 220 °C, and hold for 5 min) were adjusted, and 0.5 µl of the sample was injected (split 1:20). Pyruvate, acetic acid, and succinic acid were determined via HPLC (Elite La Chrome L-2130, L-2200, L-2300, L-2455, Hitachi) according to Sturm-Richter et al. [2]. Acetoin was measured photometrically at 490 nm using the Voges–Proskauer reaction in a 96-well assay (Sigma-Aldrich, Darmstadt, Germany). Standards were used for a calibration curve. A sample volume of 20 µl was used and mixed with the reaction buffer (100 µl H_2_O, 14 µl 10 mg/ml l-arginine, 12 µl Barritt's reagent A). The Voges–Proskauer reaction was initiated with the addition of 6 µl Barritt's reagent B and the reaction was incubated for 30 min at 37 °C.

## Results

### Characterization of *E. coli* mutants under anoxic conditions

**Fig. 3:**
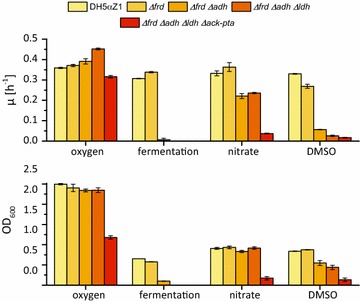
Growth analysis of the generated fermentation mutants. Genotypes: WT (DHαZ1), JG11 (Δ*frd*), JG369 (Δ*frd* Δ*adhE*), JG472 (Δ*frd* Δ*adhE* Δ*ldhA*), and JG806 (Δ*frd* Δ*adhE* Δ*ldhA* Δ*pta*–*ack*). Growth rates and final values for the optical density are shown for the deletion mutants compared to the initial strain

The goal of this study was to establish an electrode-assisted fermentation in *E. coli.* In other words, it was sought to establish a specialized anoxic respiratory metabolism in *E. coli* to reach high production efficiency and to transfer the surplus of electrons to an anode surface. In the first step, the central metabolism of *E. coli* was engineered for the accumulation of high pyruvate concentrations under anoxic condition. Compared to oxic or fermentative conditions, there is no report in the literature that would systematically analyze the impact of deletions in genes encoding central enzymes of mixed acid fermentation pathways under anoxic respiratory conditions [40]. Hence, the developed mutants were characterized regarding growth and end product accumulation in a minimal medium at 37 °C. Interestingly, deletion of the fumarate reductase genes (*frd*) caused an increased growth rate under all conditions tested, besides DMSO reduction. The additional deletion of the alcohol dehydrogenase gene (*adhE*) almost fully suppressed growth under fermentative conditions (0.007 ± 0.007/h) and had a pronounced negative impact on anoxic respiration. Of note, the cells were more affected with DMSO as electron acceptor compared to nitrate. The Δ*frd* Δ*adhE* Δ*ldhA* triple mutant was unable to grow under anoxic conditions without an electron acceptor. Furthermore, an additional effect on anoxic respiratory growth compared to the double deletion mutant was detected. Still, aerobic growth was 1.25-fold faster compared to the initial strain of this study [41]. The additional deletion of the phosphotransacetylase and acetate kinase genes (*Δack*-*pta*) led to a strain with the lowest growth rates of all strains tested. The acquired data are summarized in Fig. 3 and are shown in detail in Additional file 4: Table S4.

### Accumulation of acetic acid and pyruvate during anaerobic respiration

The production of mixed acid fermentation end products was of interest since later experiments aimed at the production of acetoin from pyruvate. Hence, the focus of this section will be on the accumulation of pyruvate and acetate in the supernatant growth medium of the individual strains. The concentrations of these two compounds and of the other typical end products of mixed acid fermentation can be found in the supplementary section (Additional file 5: Table S5).

The Δ*frd* Δ*adhE* Δ*ldhA* triple mutant accumulated high amounts of acetic acid in the medium during anaerobic respiration with DMSO, while the acetate concentration was very similar to the initial strain under nitrate-reducing conditions. Pyruvate was detected only in small amounts during anaerobic respiration (DMSO: 0.037 ± 0.025 mol pyruvate/mol glucose consumed, nitrate: 0.018 ± 0.008 mol pyruvate/mol glucose consumed). The quadruple mutant (Δ*frd* Δ*adhE* Δ*ldhA* Δ*pta*–*ack*) showed minimal production of acetic acid, while pyruvate was secreted to the medium in high concentrations. During anaerobic growth with DMSO as terminal electron acceptor, 65% of the consumed glucose was converted to pyruvate (1.3 ± 0.12 mol pyruvate/mol glucose consumed). With nitrate as electron acceptor an insignificantly higher yield of 70% (1.4 ± 0.16 mol pyruvate/mol glucose consumed) was achieved. Under oxic conditions pyruvate was secreted to the medium as well, but was consumed after the depletion of glucose (data not shown).

### Acetoin production in *E. coli* JG806 pMAL *alsSD*

**Fig. 4:**
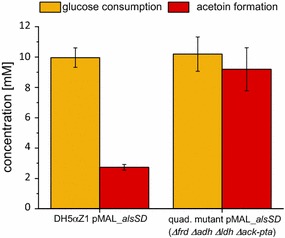
Acetoin production from glucose with nitrate as the terminal electron acceptor. The quadruple mutant carrying the vector for the acetoin production showed a strong increase in acetoin formation with nitrate as the terminal electron acceptor compared to a DH5αZ1 strain with the same vector. The acetoin yield reached a value of 0.9 mol/mol glucose consumed

The quadruple mutant strain JG806 was revealed to secrete high amounts of pyruvate to the medium with nitrate as the electron acceptor and was therefore used for further strain development. *E. coli* DH5αZ1 and *E. coli* JG806 were transformed with a pMAL plasmid carrying *alsS* and *alsD* from *Bacillus subtilis* under control of an IPTG inducible promoter. During anaerobic growth with nitrate as terminal electron acceptor, acetoin production was determined and normalized to the amount of glucose consumed. For DH5αZ1 cells, the amount of acetoin produced from glucose under nitrate-reducing conditions was below 30% of the theoretical maximum. The highest yield of acetoin was produced by the mutant strain under nitrate-reducing conditions. Approximately 90% of the theoretical maximum yield of acetoin production was achieved. In summary, the production of acetoin was increased threefold (nitrate) in strain JG806 compared to a strain with a non-engineered metabolism (Fig. 4). Detailed yields of all fermentation products can be found in Additional file 6: Table S6.

### Electrode-assisted fermentation

**Fig. 5:**
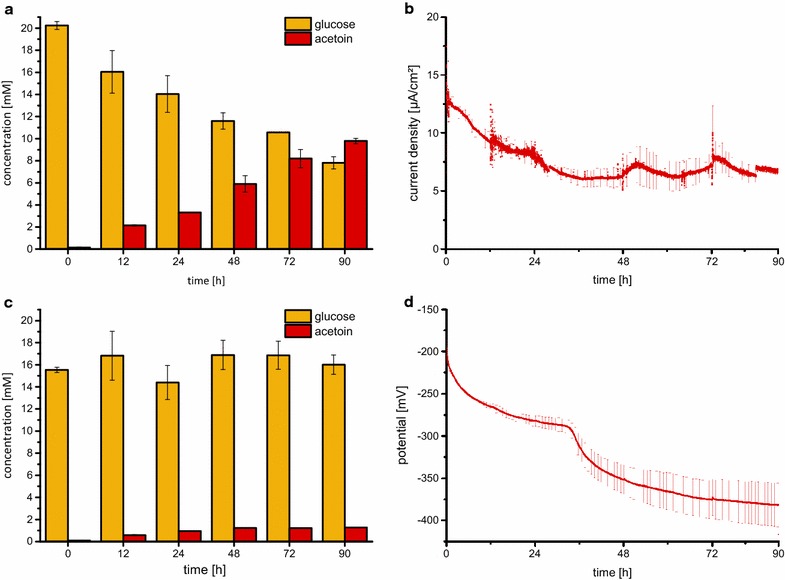
Results of the unbalanced fermentation approach. **a** Chemical analysis of the chronoamperometric experiment. The glucose concentration decreased by 12.4 ± 0.9 mM over 90 h. The acetoin concentration increased to 9.8 ± 0.2 mM after 90 h. **b** Chronoamperometric detection. The current density decreased from its maximum value of 25.7 µA/cm^2^ at the beginning and then stabilized after roughly 30 h around a value of 6 µA/cm^2^. **c** Chemical analysis of the open-circuit potentiometry experiment. No significant decrease in glucose was detected while a small amount of acetoin was formed (1.2 ± 0.03 mM). **d** Open-circuit potentiometry. The cell potential decreases in two steps from −200 to −380 mV

To prove the functionality of an electrode-assisted fermentation, the established quadruple mutant was tested in a bioelectrochemical setup. The mutant contained the necessary genes *cymA*, *mtrA*, and *stc* for an electron transport chain into the periplasm [2]. Previous research revealed that this electron transport chain can be connected to an anode of a bioelectrochemical system by using methylene blue as electron shuttle [2]. We developed a bioelectrochemical reactor with a high surface-to-volume ratio to show that an electrode-assisted acetoin production is possible. The results of these experiments are presented in Fig. 5. The detected average current density of the chronoamperometric detection was 6.2 ± 1.4 µA/cm^2^. During the fermentation, a charge of 81.6 ± 18.5 C was transferred to the anode which corresponds to 0.85 ± 0.19 mmol of electrons. The quantification of reactants showed a decrease in glucose concentration of 12.4 ± 0.92 mM within 90 h, while acetoin increased to 9.8 ± 0.2 mM. HPLC and GC measurements did not reveal any other end product besides acetoin. Hence, glucose molecules that were not converted into acetoin could be used for biomass formation, non-growth-associated metabolism (NGAM), or carbon dioxide formation within the citric acid cycle. Only the last process would be connected to a further release of electrons. Consequently, the coulombic efficiency of the process will be within a window of 20.2–93.4%. The first value accounts for a full oxidation, while the latter is based on biomass formation of/from the glucose consumed that was not converted into acetoin. The open-circuit potentiometry experiment, in which no current flow between anode and cathode was sustained by the potentiostat, showed no significant decrease in glucose concentration and only a minimal formation of acetoin to 1.2 ± 0.03 mM. The final value for the cell potential was −380 mV after 90 h, which indicates that the electron mediator methylene blue occurred almost exclusively in its reduced state under this condition.

## Discussion

Several studies analyzed the effects of single mutations in genes encoding for key enzymes of the anoxic fermentative metabolism in *E. coli* [42–44]. Nevertheless, a thorough analysis of the growth capabilities of these mutants under respiratory and fermentative conditions and a subsequent analysis of metabolic end products have so far not been conducted. Therefore, we combined this analysis with our aim to construct a strain that would accumulate pyruvate under anoxic respiratory conditions.

It was surprising to observe that deletion of the fumarate reductase and alcohol dehydrogenase encoding genes already led to a strain that was almost not capable of fermentative growth anymore, although lactate production alone would be suitable to recycle the cofactor NAD^+^. Still, even the initial strain used in this study produced lactate only in minor quantities under fermentative conditions and the regulatory routines of *E. coli* apparently do not seem to sustain a flexible production of end products steered by the available possibilities to balance the cellular redox state.

Interestingly, the deletion of *frdA*-*D* (fumarate reductase), *adhE* (alcohol dehydrogenase), and *ldhA* (lactate dehydrogenase) led to a 1.25-fold increased growth rate under aerobic and decreased growth rates under anaerobic respiratory conditions. Aerobic respiration is accompanied by the almost full oxidation of glucose to carbon dioxide (Additional file 5: Table S5). Side pathways from the canonical routine of glycolysis and citric acid cycle will therefore not lead to faster growth. Nevertheless, conditions in a shaker flask will not provide every individual cell with equivalent shares of oxygen and it seems reasonable to assume that oxygen depletion might lead in some cells to a partial switch to anoxic growth [45, 46]. This would be accompanied with the production of mixed acid fermentation end products and lower ATP yields [47]. Streamlining of the metabolism by deletion of fermentation pathways ensures that all cells have to metabolize glucose directly to carbon dioxide even if oxygen might be the limiting factor. As a consequence the cells will grow faster.

The situation is different under anoxic respiratory conditions. The absence of oxygen triggers a downregulation of the citric acid cycle, which leads to an incomplete oxidation of glucose via fermentative pathways [48]. Therefore, blockage of these pathways reduces the substrate conversion rates and as a consequence also the growth rate. Still at least under nitrate-reducing conditions, evidence was provided that some part of the growth substrate is completely oxidized to carbon dioxide via the citric acid cycle [47]. This is not possible with DMSO instead of nitrate as electron acceptor. DMSO reduction is menaquinol (*E*°′  =  −74 mV) dependent. The low midpoint redox potential of the menaquinone/menaquinol pair hampers respiratory glucose consumption coupled to DMSO reduction, because *E. coli* cannot use a reverse electron transfer-coupled succinate oxidation within the citric acid cycle. The utilization of nitrate as an electron acceptor sustains succinate oxidation within the citric acid cycle because nitrate reduction is functional with ubiquinone (*E*°′  =  +113 mV) as electron shuttle within the membrane [49, 50]. It might be this reaction within the citric acid cycle that leads to the observed differences in the phenotypes of the mutants compared to the initial strain under respiratory growth with nitrate or DMSO, respectively. Acetate is the main end product under anoxic conditions. Its production is accompanied by the formation of 1 mol ATP per mol acetate. Hence, it is not surprising that a deletion of the *ack/pta* genes negatively influenced growth of the *E. coli* strains under anoxic conditions.

The quadruple mutant Δ*frd* Δ*adhE* Δ*ldhA* Δ*pta*–*ack* produced nearly no acetic acid, but accumulated pyruvate instead with a yield of 1.4 mol pyruvate per mol of glucose, which is close the value of 1.6 mol/mol achieved by Causey and colleagues using a strain deficient of the pyruvate oxidase and the ability to gain energy by oxidative phosphorylation [28]. Therefore, this strain was selected for further development towards acetoin production. In this study, acetoin was produced with a yield of 90% with nitrate as the terminal electron acceptor. This represents the highest yield reported for anaerobic acetoin production in *E. coli* [23], indicating the efficiency of the engineered metabolism. The studies listed in Table 1 report acetoin production in several approaches. Although it is common to investigate acetoin producing strains in complex media containing significant amounts of yeast extract or other fermentable compounds, the acetoin yields are calculated in consideration of the added glucose only. Nevertheless, the literature is full of examples in which productivities over 100% are reached due to the catabolic consumption of complex media constituents [40]. It is not unlikely to assume that the achievable rates will increase if higher amounts of complex media constituents are added. To our best knowledge, the overall highest efficiency for the fermentation of acetoin was reported by Ui et al. with a value of 96% in an engineered *E. coli* strain [51]. However, the experiments have been conducted in oxic LB medium, containing 15 g/l of complex organic compounds. In this study, only 1 g/l of casamino acids was supplemented to the medium to allow for the analysis of the coulombic efficiency in the subsequent bioelectrochemical process. Works of Chen et al. and Zhang et al. conducted the fermentation process in a comparable minimal medium under oxic to microaerobic conditions with engineered strains of *B. subtilis* as the catalyst organism [22, 52]. The achieved yields reach values between 60.5 and 75%. Hence, the yields in this study range well within notable benchmarks.

**Fig. 6:**
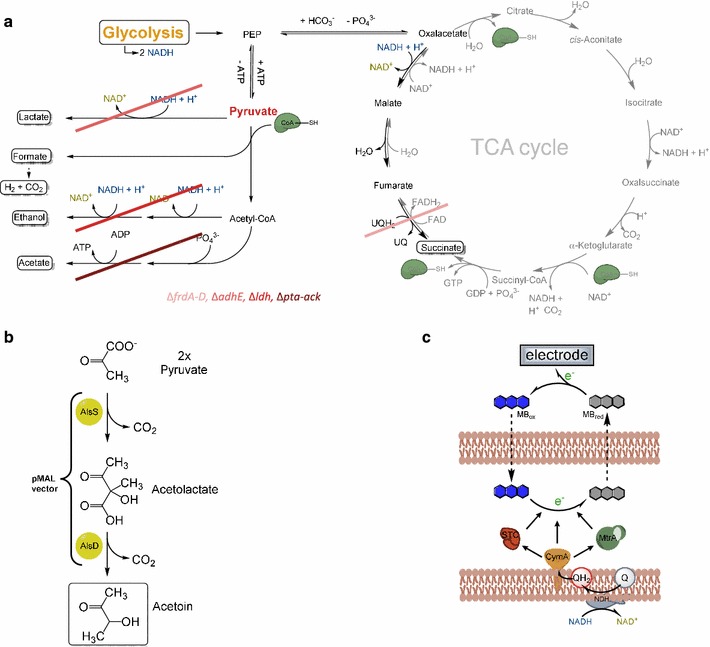
Overview of the implemented modification in the metabolism of *E. coli*. **a** The knock-out of the genes *frdA*-*D, adhE, ldh, pta*, and *ack* inhibits the regeneration of NAD^+^. Furthermore, the anaerobic catabolism is forced to stop at the stage of pyruvate. **b** Accumulated pyruvate is converted to acetoin by the acetolactate synthase (AlsS) and the acetolactate decarboxylase (AlsD), expressed from a pMAL vector. **c** The surplus of respiratory electrons in the form of NADH is transferred to a carbon electrode. The *C*-type cytochromes CymA, Stc, and MtrA from *Shewanella oneidensis* enable the export of electrons into the periplasm. The electrons, transported into the periplasm, are transferred onto *methylene blue* (MB), a membrane-diffusible redox shuttle. Reduced methylene blue is reoxidized at the carbon electrode, which serves as a non-depletable terminal electron acceptor

In the last step, the respiratory electron transport chain of *E. coli* was extended by the expression of the c-type cytochromes CymA, MtrA, and STC, facilitating a methylene blue-mediated electrode respiration. The modifications leading to this catalyst strain are summarized in Fig. 6. During the electrode-assisted fermentation, an acetoin yield of 79% was achieved. No other end product besides acetoin was detectable. The coulombic efficiency of the process was 93.4%, if it is assumed that the amount of glucose which was not converted into acetoin was used for NGAM or the formation of biomass. Biomass formation is not accompanied by the release of electrons as biomass [(CH_2_O)_n_] and glucose have the same oxidation state. A complete oxidation of the glucose molecules that were not converted into acetoin to CO_2_ seems unlikely, since the citric acid cycle—as stated above—will not be functional under the operational conditions.

Recently, Bursac et al. could show acetoin production in an analogous setup. The authors used *S. oneidensis* as biocatalyst. This organism can conduct an extracellular electron transfer to an electrode surface. This is certainly an advantage regarding the development of electrode-assisted fermentation processes. Nevertheless, the *E. coli* strain used in this study is able to metabolize a larger spectrum of carbon sources including glucose, sucrose, and glycerol. Moreover, the strain produces acetoin with a similar yield but is still able to connect this metabolism to (slow) growth. Hence, the developed *E. coli* strain can be applied in a continuous culture, while the *S. oneidensis* biocatalyst will either have to be replaced or the growth conditions would have to be changed to oxic conditions. In conclusion, both strategies for enabling electrode-assisted fermentations have their advantages, and while the *E. coli* strain must be developed further for higher electron transfer rates, *S. oneidensis* would have to be developed in the direction of a broader spectrum of useable growth substrates.

## Conclusion

In this study, a potential platform organism for pyruvate-dependent production processes was developed. It accumulates high amounts of pyruvate during anaerobic respiration to the medium broth. The presented process for acetoin production is so far unique and offers the potential for a number of newly efficient biotechnological production routines. Of note, all electrode-assisted fermentations are accompanied with the production of an electrical current as side product. This current can be used to sustain the process or to produce hydrogen on the cathode site via a microbial electrolysis cell approach. We do not want to conceal that we can achieve far high yields but that the production rates have to be improved. Further experiments will have to tackle this limitation, for instance, via random mutagenesis and selection for higher substrate conversion rates.

## Additional files

**Additional file 1: Table S1.** Oligonucleotides used in this study.

**Additional file 2: Table S2.** Plasmids used in this study.

**Additional file 3: Table S3.** Bacterial strains used in this study.

**Additional file 4: Table S4.** Summary of growth experiments: µ and final optical density.

**Additional file 5: Table S5.** Summary of growth experiments: end products.

**Additional file 6: Table S6.** Comparison of end-product yields in DH5αZ1 and JG806 expressing pMAL_*alsSD* with NO_3_^−^ as the electron acceptor.
