# Supplementary material for: Electrode-assisted acetoin production in a metabolically engineered Escherichia coli strain
Source: Biotechnol Biofuels. 2017 Mar 14;10:65. doi: 10.1186/s13068-017-0745-9 (PMC5348906; doi:10.1186/s13068-017-0745-9)
Supplement: Supplementary file 1 — Additional file 1: Table S1. Oligonucleotides used in this study. [file 13068_2017_745_MOESM1_ESM.docx]

Table S 1: Oligonucleotides used in this study.

| No. | Sequence |
| --- | --- |
| 1 | 5’‑ATCGGCATTGCCCAGAAGGGGCCGTTTATGTTGCCAGACAGCGCTACTGAAATGCTCTCCTGATAATGTTAAACTTTTTTAGTAAATCATCTGCTCGAATGCTTAGTACGTACTATCAACA |
| 2 | 5’‑ATGGCTGTTACTAATGTCGCTGAACTTAACGCACTCGTAGAGCGTGTAAAAGAATACAAGCTTGCATGCC |
| 3 | 5’‑TGTTGTGGAAGCCGTTATAG |
| 4 | 5’‑CATAGGTTAAGCAAATCATCAC |
| 5 | 5’‑TAACTGAGAAGAGCTAGGCAATCAACGGTATAGTATCTCAGGTCGAGCTCTTCATTTCGTCTATATTGCTGAAGGTAC |
| 6 | 5’‑AGTAGCTTAAATGTGATTCAACATCACTGGAGAAAGTCTTGCCAGTTGGCATGTTTTACC |
| 7 | 5’‑CGTGTGATTCGATAGCTCTTCACTGCGCTGTAGCGAACAGTCAC |
| 8 | 5’‑TACAGTCAGCATTCAGCGCTCTTCGAAAGGCGAAACCTGCCCG |
| 9 | 5’‑CGTGATGCTAGCTAGTTGATCTAGATGACGCTGAAGAGCCCGATGAGTAGCCCTGAGATATTGCCTAGCTCTTCACTTGCCAGTTGGCATGTTTTACC |
| 10 | 5’‑CCACTCTACATCGATCACTCTAGAACACGCTGTAGCGAACAGTCAC |
| 11 | 5’‑ATGGTAGTTAGTAGTGTAGACATGGCTCTTCGAAGACTTTCTCCAGTGATGTTGAA |
| 12 | 5’‑TCGATCGATCGACTACACGTCAGCTCTTCTCGCCCTGATAACGCAGTTG |
| 13 | *5’‑CATGTGGGAGTTTATTCTTG |
| 14 | 5’‑TTCGACAAAAATCTAGAAATAATTTTGTTTAACTTTAAGAAGGAGATATACAAAAGCTTTCAGCACTGTCCTGCTCC |
| 15 | 5’‑ATGTGACTGAGCTAGCATTACCCTGTTATCCCTACGATCGGCGGCATAAAAC |
| 16 | *5’‑GGAAGTACCTATAATTGATAC |
| 17 | 5’‑TAAAGAGACTTTTTACGTTTGTAAACCATCACAAGGAGCAGGACAGTGCTGATCTCGTCATCATCCGCAG |
| 18 | 5’‑AGACAAAAAAAATGTCGCACAATGTGCGCCATTTTTCACTTCACAGGTCAATTACCCTGTTATCCCTAGATCCTGAGGTTAATCCTTC |
| 19 | 5’‑GGTAAACTTAAGGCGAACAG |
| 20 | 5’‑TTATCAATGGTTCCTGTACG |
| 21 | 5’‑ GCATAATAAGGAGTGAGGGTG |
| 22 | 5’‑ AAAATCAGGAAAAGAGCCAAAT |

*phosphorylated at 5’-end
