# Supplementary material for: Electrode-assisted acetoin production in a metabolically engineered Escherichia coli strain
Source: Biotechnol Biofuels. 2017 Mar 14;10:65. doi: 10.1186/s13068-017-0745-9 (PMC5348906; doi:10.1186/s13068-017-0745-9)
Supplement: Supplementary file 2 — Additional file 2: Table S2. Plasmids used in this study. [file 13068_2017_745_MOESM2_ESM.docx]

Table S 2: Plasmids used in this study.

| plasmid | genotype | reference |
| --- | --- | --- |
| pAH95_stc | KanR, oriR6K, attP21, P_ara_, stc | [2] |
| pASK43+ | Amp^R^, f1 Origin | IBA, Göttingen |
| pASK43+csc_ldh-RecSites | pASK43+, *cscRAKB*, homologous sequences for *ldhA*-deletion in *E. coli* | this study |
| pASK43+_pta-ack_Del_SceI | pASK43+, *galK*, homologous sequences for *pta‑ack*‑deletion in *E. coli* | this study |
| pEC86 | ChlR, ccmABCDEFGH, P_tet_ | [37] |
| pKD46 | Amp^R^, [tL3], oriR101, P_araB_ λ Red Gene^Ara^ | [33] |
| pMAL_alsSD | Amp^R^, P_lac_ *alsSD* | this study |
| pKJL 124 | Chl^R^ *csc^+^* *cscKo124* | [54] |
| pSG76-CSH | Chl^R^, sourrounded with two I-SceI-recognition sites | [32] |
| pSTKST | Kan^R^, I-SceI^tet^ | [32] |
