# Supplementary material for: Electrode-assisted acetoin production in a metabolically engineered Escherichia coli strain
Source: Biotechnol Biofuels. 2017 Mar 14;10:65. doi: 10.1186/s13068-017-0745-9 (PMC5348906; doi:10.1186/s13068-017-0745-9)
Supplement: Supplementary file 3 — Additional file 3: Table S3. Bacterial strains used in this study. [file 13068_2017_745_MOESM3_ESM.docx]

Table S 3: Bacterial strains used in this study.

| strain | genotype | reference |
| --- | --- | --- |
| JG22 (DH5αZ1) | aci^q^, PN25-tetR, Sp^R^, deoR, supE44, Δ (lacZYA-argFV169), Phi80 lacZΔM15 | [55] |
| JG146 | DH5αZ1 Δ (napC–F) Δ (frdA–D) 4380508:: (Ptet cymA-mtrA) | [31] |
| JG187 | JM 109 pKJL 124 | laboratory collection |
| JG287 | JG146 Δ (*napC-F*) (*frdA-D*) 4380508:: (P_tet_ *cymA-mtrA*) Δ*galK*, Δ (*gspC-gspO*)::CP6-*galK* | laboratory collection |
| JG369 | JG146 Δ*adhE* | this study |
| JG472 | JG369 ΔldhA ΔgalK | this study |
| JG479 | JG144  pASK43+csc_ldh-RecSites | this study |
| JG806 | JG472 Δpta-ack::galK | this study |
| JG613 | *Bacillus subitilis* PY79 | laboratory collection |
| JG991 | JG806 P21::pAH95_stc, pEC 86 pMAL *alsSD* | this study |
| JM 109 | *end*A1, *rec*A1, *gyr*A96, *thi*, *hsd*R17  (r_k_^–^, m_k_^+^), *rel*A1, *sup*E44, Δ (*lac‑pro*AB), [F´ *tra*D36, *pro*AB, *laq*I^q^ZΔM15] | Promega, Madison, USA |
