# Supplementary material for: Electrode-assisted acetoin production in a metabolically engineered Escherichia coli strain
Source: Biotechnol Biofuels. 2017 Mar 14;10:65. doi: 10.1186/s13068-017-0745-9 (PMC5348906; doi:10.1186/s13068-017-0745-9)
Supplement: Supplementary file 4 — Additional file 4: Table S4. Summary of growth experiments: µ and final optical density. [file 13068_2017_745_MOESM4_ESM.docx]

Table S 4: Summary of growth experiments: µ and final optical density.

| Strain | Genotype | Oxygen | | Fermentation | | Anaerobic respiration  (nitrate) | | Anaerobic respiration (DMSO) | |
| --- | --- | --- | --- | --- | --- | --- | --- | --- | --- |
|  |  | µ | OD | µ | OD | µ | OD | µ | OD |
| JG22 | non-modified | 0.359  ±  0.003 | 2.498  ±  0.017 | 0.307  ±  0.001 | 0.650  ±  0.000 | 0.332  ±  0.012 | 0.908  ±  0.026 | 0.330  ±  0.002 | 0.838  ±  0.003 |
| JG11 | *Δfrd* | 0.371  ±  0.005 | 2.403  ±  0.090 | 0.338  ±  0.003 | 0.573  ±  0.003 | 0.363  ±  0.022 | 0.933  ±  0.033 | 0.369  ±  0.010 | 0.875  ±  0.006 |
| JG369 | *Δfrd adhE* | 0.391  ±  0.013 | 2.340  ±  0.034 | 0.007  ±  0.007 | 0.098  ±  0.003 | 0.221  ±  0.012 | 0.833  ±  0.026 | 0.057  ±  0.001 | 0.547  ±  0.059 |
| JG472 | *Δfrd adhE ldhA* | 0.452  ±  0.004 | 2.348  ±  0.057 | -- | -- | 0.236  ±  0.003 | 0.916  ±  0.030 | 0.026  ±  0.003 | 0.439  ±  0.052 |
| JG806 | *Δfrd adhE ldhA pta-ack* | 0.316  ±  0.006 | 1.180  ±  0.042 | -- | -- | 0.037  ±  0.002 | 0.172  ±  0.041 | 0.017  ±  0.002 | 0.134  ±  0.040 |
