# Supplementary material for: Electrode-assisted acetoin production in a metabolically engineered Escherichia coli strain
Source: Biotechnol Biofuels. 2017 Mar 14;10:65. doi: 10.1186/s13068-017-0745-9 (PMC5348906; doi:10.1186/s13068-017-0745-9)
Supplement: Supplementary file 5 — Additional file 5: Table S5. Summary of growth experiments: end products. [file 13068_2017_745_MOESM5_ESM.docx]

Table S 5: Summary of growth experiments: end products.

| Strain | Acceptor | Pyruvate | | Acetic acid | | Succinic acid | | Lactic acid | | Formic acid | | Ethanol | | C‑recovery  % |
| --- | --- | --- | --- | --- | --- | --- | --- | --- | --- | --- | --- | --- | --- | --- |
|  |  | mol/mol | % | mol/mol | % | mol/mol | % | mol/mol | % | mol/mol | % | mol/mol | % |  |
| WT | O_2_ | 0.002 ± 0.000 | 0.1 ± 0.0 | 0.006 ± 0.005 | 0.3 ± 0.3 | 0.052 ± 0.006 | 2.6 ± 0.3 | 0.018 ± 0.005 | 0.9 ± 0.3 | 0.006 ± 0.001 | 0.3 ± 0.1 | 0.000 ± 0.000 | 0.0 ± 0.0 | 4.7 ± 0.6 |
|  | -- | 0.002 ± 0.002 | 0.1 ± 0.1 | 0.675 ± 0.121 | 33.8 ± 6.1 | 0.124 ± 0.018 | 6.2 ± 0.9 | 0.014 ± 0.002 | 0.7 ± 0.1 | 0.284 ± 0.038 | 14.2 ± 1.9 | 0.847 ± 0.187 | 42.4 ± 9.4 | 64.5 ± 11.9 |
|  | DMSO | 0.015 ± 0.010 | 0.8 ± 0.5 | 0.839 ± 0.055 | 42.0 ± 2.8 | 0.152 ± 0.010 | 7.6 ± 0.5 | 0.032 ± 0.011 | 1.6 ± 0.6 | 0.002 ± 0.000 | 0.1 ± 0.0 | 0.640 ± 0.049 | 32.0 ± 2.5 | 61.8 ± 3.9 |
|  | NO_3_^-^ | 0.012 ± 0.002 | 0.6 ± 0.1 | 0.707 ± 0.057 | 35.4 ± 2.9 | 0.196 ± 0.011 | 9.8 ± 0.6 | 0.075 ± 0.019 | 3.8 ± 1.0 | 0.527 ± 0.047 | 26.4 ± 2.4 | 0.000 ± 0.000 | 0.0 ± 0.0 | 49.8 ± 3.6 |
| JG11 | O_2_ | 0.004 ± 0.000 | 0.2 ± 0.0 | 0.003 ± 0.002 | 0.2 ± 0.1 | 0.002 ± 0.002 | 0.1 ± 0.1 | 0.003 ± 0.001 | 0.2 ± 0.1 | 0.009 ± 0.001 | 0.5 ± 0.1 | 0.000 ± 0.000 | 0.0 ± 0.0 | 0.7 ± 0.2 |
|  | -- | 0.000 ± 0.000 | 0.0 ± 0.0 | 0.757 ± 0.026 | 37.9 ± 1.3 | 0.032 ± 0.002 | 1.6 ± 0.1 | 0.010 ± 0.001 | 0.5 ± 0.1 | 0.289 ± 0.026 | 14.5 ± 1.3 | 0.907 ± 0.014 | 45.4 ± 0.7 | 62.1 ± 0.6 |
|  | DMSO | 0.000 ± 0.000 | 0.0 ± 0.0 | 0.908 ± 0.045 | 45.4 ± 2.3 | 0.064 ± 0.013 | 3.2 ± 0.7 | 0.003 ± 0.001 | 0.2 ± 0.1 | 0.000 ± 0.000 | 0.0 ± 0.0 | 0.749 ± 0.052 | 37.5 ± 2.6 | 59.7 ± 1.3 |
|  | NO_3_^-^ | 0.005 ± 0.001 | 0.3 ± 0.1 | 0.651 ± 0.065 | 32.5 ± 3.3 | 0.081 ± 0.011 | 4.1 ± 0.6 | 0.075 ± 0.005 | 3.8 ± 0.3 | 0.013 ± 0.004 | 0.7 ± 0.2 | 0.051 ± 0.016 | 2.6 ± 0.8 | 32.9 ± 3.2 |
| JG369 | O_2_ | 0.000 ± 0.000 | 0.0 ± 0.0 | 0.014 ± 0.004 | 0.7 ± 0.2 | 0.028 ± 0.018 | 1.4 ± 0.9 | 0.008 ± 0.006 | 0.4 ± 0.3 | 0.007 ± 0.003 | 0.4 ± 0.2 | 0.000 ± 0.000 | 0.0 ± 0.0 | 2.9 ± 0.9 |
|  | -- | 0.003 ± 0.003 | 0.2 ± 0.2 | 0.191 ± 0.040 | 9.6 ± 2.0 | 0.179 ± 0.035 | 9.0 ± 1.8 | 1.082 ± 0.093 | 54.2 ± 4.7 | 0.411 ± 0.101 | 20.6 ± 5.1 | 0.018 ± 0.001 | 0.9 ± 0.1 | 79.9 ± 9.0 |
|  | DMSO | 0.008 ± 0.007 | 0.4 ± 0.4 | 1.027 ± 0.036 | 51.4 ± 1.8 | 0.090 ± 0.004 | 4.5 ± 0.2 | 0.424 ± 0.049 | 21.2 ± 2.5 | 0.001 ± 0.001 | 0.1 ± 0.1 | 0.005 ± 0.003 | 0.3 ± 0.2 | 62.0 ± 3.7 |
|  | NO_3_^-^ | 0.003 ± 0.001 | 0.2 ± 0.1 | 0.505 ± 0.033 | 25.3 ± 1.7 | 0.037 ± 0.003 | 1.9 ± 0.2 | 0.133 ± 0.008 | 6.7 ± 0.4 | 0.694 ± 0.059 | 34.7 ± 3.0 | 0.000 ± 0.000 | 0.0 ± 0.0 | 37.7 ± 2.1 |
| JG472 | O_2_ | 0.017 ± 0.002 | 0.9 ± 0.1 | 0.005 ± 0.005 | 0.3 ± 0.1 | 0.002 ± 0.002 | 0.1 ± 0.1 | 0.017 ± 0.002 | 0.9 ± 0.1 | 0.007 ± 0.001 | 0.4 ± 0.1 | 0.000 ± 0.000 | 0.0 ± 0.0 | 1.4 ± 0.4 |
|  | -- | -- | -- | -- | -- | -- | -- | -- | -- | -- | -- | -- | -- | -- |
|  | DMSO | 0.037 ± 0.025 | 1.9 ± 1.3 | 1.504 ± 0.047 | 75.2 ± 2.4 | 0.097 ± 0.009 | 4.9 ± 0.5 | 0.037 ± 0.025 | 1.9 ± 1.3 | 0.003 ± 0.002 | 0.2 ± 0.1 | 0.009 ± 0.004 | 0.5 ± 0.2 | 59.6 ± 0.9 |
|  | NO_3_-^-^ | 0.018 ± 0.008 | 0.9 ± 0.4 | 0.655 ± 0.025 | 32.8 ± 1.3 | 0.059 ± 0.009 | 3.0 ± 0.5 | 0.018 ± 0.008 | 0.9 ± 0.4 | 0.542 ± 0.198 | 27.1 ± 9.9 | 0.000 ± 0.000 | 0.0 ± 0.0 | 36.0 ± 2.4 |
| JG806 | O_2_ | 0.445 ± 0.063 | 22.3 ± 3.2 | 0.314 ± 0.028 | 15.7 ± 1.4 | 0.051 ± 0.009 | 2.6 ± 0.5 | 0.081 ± 0.013 | 4.1 ± 0.6 | 0.007 ± 0.001 | 0.4 ± 0.1 | 0.047 ± 0.030 | 2.4 ± 1.5 | 41.8 ± 2.4 |
|  | -- | -- | -- | -- | -- | -- | -- | -- | -- | -- | -- | -- | -- | -- |
|  | DMSO | 1.301 ± 0.121 | 65.1 ± 6.1 | 0.058 ± 0.006 | 2.9 ± 0.3 | 0.053 ± 0.012 | 2.7 ± 0.6 | 0.026 ± 0.003 | 1.3 ± 0.2 | 0.011 ± 0.001 | 0.6 ± 0.1 | 0.032 ± 0.009 | 1.6 ± 0.5 | 73.1 ± 5.8 |
|  | NO_3_-^-^ | 1.414 ± 0.160 | 70.7 ± 8.0 | 0.087 ± 0.039 | 4.4 ± 1.9 | 0.023 ± 0.023 | 1.2 ± 1.2 | 0.075 ± 0.044 | 3.8 ± 2.2 | 0.015 ± 0.005 | 0.8 ± 0.3 | 0.001 ± 0.001 | 0.1 ± 0.1 | 79.2 ± 6.6 |
