# Supplementary material for: Electrode-assisted acetoin production in a metabolically engineered Escherichia coli strain
Source: Biotechnol Biofuels. 2017 Mar 14;10:65. doi: 10.1186/s13068-017-0745-9 (PMC5348906; doi:10.1186/s13068-017-0745-9)
Supplement: Supplementary file 6 — Additional file 6: Table S6. Comparison of end-product yields in DH5αZ1 and JG806 expressing pMAL_alsSD with NO3 − as the electron acceptor. [file 13068_2017_745_MOESM6_ESM.docx]

Table S 6: Comparison of end-product yields in DH5αZ1 and JG806 expressing pMAL_*alsSD* with NO_3_^-^ as the electron acceptor.

| product  strain | | DH5αZ1 pMAL_*alsSD* | JG806 pMAL_*alsSD* |
| --- | --- | --- | --- |
| Pyruvic acid | mol/mol | 0,000 ± 0,000 | 0,000 ± 0,000 |
|  | Yield % | 0,0 ± 0,0 | 0,0 ± 0,0 |
| Acetic acid | mol/mol | 1,005 ± 0,062 | 0,000 ± 0,000 |
|  | Yield % | 50,3 ± 3,1 | 0,0 ± 0,0 |
| Succinic acid | mol/mol | 0,110 ± 0,021 | 0,077 ± 0,023 |
|  | Yield % | 5,5 ± 1,1 | 3,9 ± 1,2 |
| Lactic acid | mol/mol | 0,006 ± 0,003 | 0,024 ± 0,011 |
|  | Yield % | 0,3 ± 0,2 | 1,2 ± 0,6 |
| Formic acid | mol/mol | 0,014 ± 0,012 | 0,002 ± 0,002 |
|  | Yield % | 0,7 ± 0,6 | 0,1 ± 0,1 |
| ethanol | mol/mol | 0,136 ± 0,027 | 0,045 ± 0,005 |
|  | Yield % | 6,8 ± 1,4 | 2,3 ± 0,3 |
| acetoin | mol/mol | 0,278 ± 0,040 | 0,898 ± 0,059 |
|  | Yield % | 27,8 ± 4,0 | 89,8 ± 5,9 |
| C-recovery in % without CO_2_ | | 64,4 ± 3,7 | 67,7 ± 5,6 |
| C-recovery in % with CO_2_ | | 73,7 ± 4,4 | 97,6 ± 7,5 |
